# Nutraceutical agents with anti-inflammatory properties prevent dietary saturated-fat induced disturbances in blood–brain barrier function in wild-type mice

**DOI:** 10.1186/1742-2094-10-73

**Published:** 2013-06-19

**Authors:** Ryusuke Takechi, Menuka M Pallebage-Gamarallage, Virginie Lam, Corey Giles, John C Mamo

**Affiliations:** 1 School of Public Health, Curtin Health Innovation Research Institute, Biosciences Research Precinct, Faculty of Health Sciences, Curtin University, Kent st, Bentley, WA, 6102, Australia; 2 Centre for Metabolic Fitness, Australian Technology Network, GPO Box U1987, Perth, WA, 6845, Australia

**Keywords:** Garlic extract-aged, Alpha-lipoic acid, Blood–brain barrier, Inflammation, Neurodegenerative disorders, Niacin, Nicotinamide, Oxidative stress, Saturated fatty acids

## Abstract

**Background:**

Emerging evidence suggests that disturbances in the blood–brain barrier (BBB) may be pivotal to the pathogenesis and pathology of vascular-based neurodegenerative disorders. Studies suggest that heightened systemic and central inflammations are associated with BBB dysfunction. This study investigated the effect of the anti-inflammatory nutraceuticals garlic extract-aged (GEA), alpha lipoic acid (ALA), niacin, and nicotinamide (NA) in a murine dietary-induced model of BBB dysfunction.

**Methods:**

C57BL/6 mice were fed a diet enriched in saturated fatty acids (SFA, 40% fat of total energy) for nine months to induce systemic inflammation and BBB disturbances. Nutraceutical treatment groups included the provision of either GEA, ALA, niacin or NA in the positive control SFA-group and in low-fat fed controls. Brain parenchymal extravasation of plasma derived immunoglobulin G (IgG) and large macromolecules (apolipoprotein (apo) B lipoproteins) measured by quantitative immunofluorescent microscopy, were used as markers of disturbed BBB integrity. Parenchymal glial fibrillar acidic protein (GFAP) and cyclooxygenase-2 (COX-2) were considered in the context of surrogate markers of neurovascular inflammation and oxidative stress. Total anti-oxidant status and glutathione reductase activity were determined in plasma.

**Results:**

Brain parenchymal abundance of IgG and apoB lipoproteins was markedly exaggerated in mice maintained on the SFA diet concomitant with significantly increased GFAP and COX-2, and reduced systemic anti-oxidative status. The nutraceutical GEA, ALA, niacin, and NA completely prevented the SFA-induced disturbances of BBB and normalized the measures of neurovascular inflammation and oxidative stress.

**Conclusions:**

The anti-inflammatory nutraceutical agents GEA, ALA, niacin, or NA are potent inhibitors of dietary fat-induced disturbances of BBB induced by systemic inflammations.

## Background

Accumulating evidence suggests that disturbances in cerebral capillary integrity characterized by inflammation, loss of blood–brain barrier (BBB) functions, parenchymal extravasation of plasma proteins, proteinaceous deposits on extracellular matrices, and glial cell activation contribute to the onset or progression of a number of neurodegenerative disorders including vascular dementia, Alzheimer’s disease, Parkinson’s disease, and multiple sclerosis [1]. Cerebral capillary abnormalities that precede frank pathological or clinical abnormalities include capillary endothelial cell proliferation, perivascular gliosis, and progressive arteriopathy, and thereafter the formation of lacunar lesions [2-5].

Cerebral capillary dysfunction induced as a consequence of a chronically heightened state of systemic inflammation is positively associated with neurovascular degenerative disorders [6-9]. Comorbidities such as dyslipidemia, hypertension or endocrine disorders, exposure to pollutants such as smoking, alcohol consumption, or atherogenic diets may also increase neurodegenerative disease onset via a cerebral capillary axis [3,6,8-10]. Studies in animal models support the latter. Wild-type mice chronically fed pro-inflammatory diets enriched in saturated fatty acids (SFA) or cholesterol showed hallmark features of BBB dysfunction including cerebral extravasation of large plasma proteins (immunoglobulin G (IgG) and macromolecules (apolipoprotein (apo) B lipoproteins)) concomitant with diminished endothelial tight junction proteins [9,11-13].

Several *in vivo* and *in vitro* studies suggest that pharmacological agents with anti-inflammatory or anti-oxidative activity may positively regulate BBB integrity through regulation of systemic inflammatory pathways [14-16]. The potent antioxidant, probucol, preserved BBB function and profoundly attenuated astroglial cell activation in SFA-fed mice [17]. The cholesterol-lowering 3-hydroxy-methyl-Co-A reductase inhibitor, atorvastatin, prevented BBB disturbances in spontaneously hypertensive rats [18]. Atorvastatin, pravastatin, and the non-selective cyclooxygenase inhibitor, ibuprofen, were shown to also restore BBB function in mice with BBB dysfunction induced by chronic ingestion of a pro-atherogenic diet [18].

Bioactive anti-inflammatories may also confer benefit to cerebral capillary vessels [19,20]. A bitter melon-attenuated dietary-fat induced BBB disturbances in wild-type mice concomitant with a significant reduction in both central and systemic inflammatory/oxidative stress markers [7]. However, paradoxical observations have been reported in other studies. Provision of omega-3 fatty acids to mice that had been maintained on an SFA enriched diet for twelve weeks, exacerbated measures of BBB dysfunction [11]. A study by Mustata et al. also showed increased glycoxidation in tendon, aorta, and plasma by anti-oxidative green tea and vitamins C and E in a diabetic rat model [21]. Soy isoflavones, such as genistein, inhibit the lipid peroxidation only with super-physiological concentrations due to its poor peroxyl radical scavenging function [22].

In the current study, we investigated the efficacy of selected anti-oxidative nutraceuticals, namely garlic extract-aged (GEA), alpha-lipoic acid (ALA), niacin, and nicotinamide (NA), in an established dietary-induced model of BBB disruption. The GEA contains anti-oxidative phytochemicals including S-allylcysteine, S-allylmercaptocysteine, diallyl sulfide, and allicin, which can scavenge reactive oxygen species. The GEA was shown to inhibit lipid peroxidation and reduce expression of key inflammatory proteins such as nuclear factor-kappa B *in vivo* and *in vitro*[23-25]. In clinical studies, GEA supplementation was found to reduce measures of oxidative stress and showed therapeutic benefits in subjects with Parkinson’s and Alzheimer’s disease [26-31]. ALA is a naturally occurring anti-oxidant that sequesters free radicals. ALA acts indirectly to enhance cellular anti-oxidant status by stimulating uptake of exogenous anti-oxidants and enhancing the production of endogenous anti-oxidant enzymes. ALA also inhibits the production of pro-inflammatory cytokines [32]. In a traumatic brain injury model, Toklu et al. reported that ALA improved BBB function concomitant with attenuated central and systemic inflammation [33]. Niacin and NA are classified within the B3 group of vitamins; however, niacin also has significant lipid-lowering effects [34]. Both niacin and NA can be converted into nicotinamide adenine dinucleotide or nicotinamide adenine dinucleotide phosphate, key mediators in enzymatic anti-oxidative reactions [34]. Morris et al. reported that niacin retards the cognitive decline in Alzheimer’s disease and, consistent with those findings, NA was found to delay the cognitive decline in Alzheimer’s subjects and reduce amyloid deposition in amyloid transgenic mice [35].

## Materials and methods

### Animals and diets

Wild-type C57BL/6J female mice were purchased from Animal Resources Centre, WA, Australia. The low-fat (LF) control chow was the maintenance diet (AIN-93M, Specialty Feeds, WA, Australia) containing < 4% (w/w) polyunsaturated fats and was free of SFA or dietary cholesterol. Mice with dietary-induced BBB dysfunction were fed a semi-synthetic diet enriched in SFA (SF07-050, Specialty Feeds, WA, Australia) containing 40% energy of fat derived from cocoa butter as previously described (5% w/w palmitic 16:0, 7% stearic 18:0) [17]. The nutraceutical supplements GEA (Kyolic, USA), ALA, niacin, or NA (all Sigma-Aldrich, USA) were incorporated into either the LF or SFA diets at a concentration of 3% (w/w), 0.2% (w/w), 1% (w/w), or 0.3% (w/w), respectively. The dose of nutraceutical ingested relative to weight was within clinical recommendations and similar to previous studies [23,26-28,30-35]. The components of GEA are standardized by the manufacturer relative to the amount of S-allylcysteine [36]. Animals were kept in individually ventilated cages with 12 h light/dark cycle, under controlled temperature (21°C) and air pressure. All animals had *ad libitum* access to the diets and water. All the procedures described in this study were approved by NHMRC accredited Curtin Animal Ethics Committee (approval no. N34-10).

### Dietary/nutraceutical intervention and sample collection

Eight mice were randomly allocated at six weeks of age to dietary or nutraceutical treatment groups of LF control chow; LF diet supplemented with GEA (LF+GEA); LF diet supplemented with ALA (LF+ALA); LF diet supplemented with niacin (LF+Niacin); LF diet supplemented with NA (LF+NA); diet enriched in SFA; SFA diet supplemented with GEA (SFA+GEA); SFA diet supplemented with ALA (SFA+ALA); SFA diet supplemented with niacin (SFA+Niacin); or SFA diet supplemented with NA (SFA+NA). After nine months of dietary/nutraceutical intervention, mice were anesthetized with pentobarbital (45 mg/kg). Blood samples were collected from a cardiac puncture, and plasma was isolated and stored at -80°C until next use. The brain was carefully removed and fixed in 4% paraformaldehyde (w/v in PBS, pH 7.2) for 24 h. Following cryoprotection with 20% sucrose for 72 h at 4°C, the brain tissues were frozen in dry ice/isopentane and stored at -80°C until next use.

### Assessment of BBB integrity

The integrity of the cerebrovascular BBB was assessed by an established method of cerebral plasma protein extravasation that indicates the unspecific blood-to-brain leakage of plasma proteins and macromolecules [9,11,12,17,18,37,38]. Briefly, 3-D quantitative immunomicroscopy was used to assess brain parenchymal abundance of IgG (Mw 155 kDa) and of apoB lipoproteins (molecular weight > 2×10^7^ kDa). Briefly, 20 μm thick cryosections were prepared from the right hemisphere of frozen brain tissues. After blocking with 10% goat serum for 30 min, the sections were incubated with goat anti-mouse IgG conjugated to Alexa488 (1:200, Invitrogen, US) for 20 h at 4°C. For the immunodetection of apoB, the sections were incubated with rabbit anti-mouse apoB (1:400, Abcam) for 20 h at 4°C, followed by an incubation with anti-rabbit IgG secondary antibody conjugated to Alexa488 (1:200, Invitrogen) for 2 h at 20°C. Following DAPI nuclei counterstaining, the sections were observed under a fluorescent microscope (Axiovert 200M, Zeiss, Germany). A positive control of BBB dysfunction from mice euthanized with carbon dioxide was utilized.

### Immunomicroscopic detection of cerebral inflammation

Neuronal inflammation was assessed by determining the abundance of parenchymal glial-fibrillar acidic protein (GFAP) and cyclo-oxygenase-2 (COX-2) using a 3-D quantitative immunofluorescent method previously described [12,17]. Briefly, 20 μm thick cryosections of the right brain hemisphere were prepared. Non-specific biding sites were blocked with 10% goat serum in PBS for 30 min at 20°C. Either rabbit anti-mouse GFAP (1:200, Abcam, UK) or rabbit anti-mouse COX-2 (1:200, Abcam) was applied to the sections for 20 h at 4°C. The sections were then incubated with goat anti-rabbit IgG conjugated with Alexa488 (1:200, Invitrogen) for 2 h at 20°C. DAPI was used to counterstain the nuclei.

### 3-D quantitative immunomicroscopy

Immunofluorescent micrographs were quantitatively analyzed in a 3-D context as previously described [12,17]. One section per animal was used in the estimated stereotaxic areas of 1.7 mm interaural and -2.1 mm Bregma. Immunofluorescent micrographs were captured with mRM digital camera (Zeiss) attached to Axiovert 200M. At a magnification of 200× (20× Zeiss Plan-Neofluar objective with 10× mRM camera), a minimum of five and three 3-D images was captured from randomly selected areas of the cortex and hippocampal formation, respectively, utilizing the AxioVision imaging software (Zeiss). The random images capture process represents approximately half of the cortex and hippocampal formation region of each section. Each 3-D image consisted of 12 Z-stack 2-D images, and the distance between the Z-stack images were 1.225 μm optimized based on Nyquist overlap theory. The voxel intensity of fluorescent dye was then analyzed in 3-D with Volocity 6.2 image analysis software (PerkinElmer, UK). Means of total fluorescent intensities of all the images in cortex and hippocampal formation regions were calculated within each animal, and thereafter compared between the groups.

### Plasma oxidative markers

Plasma anti-oxidative status was assessed by determining the plasma concentration of glutathione reductase and plasma total anti-oxidant status (TAS) using commercially available colorimetric assays (Randox, UK). The details are as described in the manufacturer’s instructions but with minor modifications. Briefly, for the glutathione reductase assay, 4 μL of plasma sample was mixed with 100 μL of substrate buffer and incubated at 37°C. Exactly at 1, 2, 3, 4, and 5 min after adding 20 μL of NADPH solution, the optical absorbance was read at 340 nm. By plotting the absorbance values, the slope of the linear portion of the curve was determined. The glutathione reductase activity was calculated by determining the reaction rate at 340 nm with the NADPH extinction coefficient of 0.00622/μM/cm.

The TAS assay measures the anti-oxidative status of the samples by measuring the suppression of radical cation 2,2′-azino-di-[3-ethylbenzthiazoline sulphonate] (ABTS) production. Briefly, 2 μL of water, standard or samples were incubated with 100 μL of chromogen solution at 37°C, and the absorbance was read at 600 nm (A1). At exactly 3 min after adding 20 μL of substrate buffer, the absorbance was read at 600 nm (A2). The suppression of ABTS formation (A2 – A1) was determined with the known value of the standard (2.14 mmol/L).

### Plasma lipids

Commercially available colorimetric assays were used to measure the concentrations of plasma cholesterol, triglycerides (Randox, UK), and non-esterified fatty acids (NEFA) (WAKO, Japan) according to the manufacturer’s instruction with some minor modifications.

Briefly, for cholesterol and triglyceride assays, 2 μL of plasma samples or standards were loaded to a 96-well microplate; 200 μL of reaction solution was then added and incubated for 5 min at 37°C. The optical absorbance was read at 550 nm. For the NEFA assay, 7 μL of plasma samples or standards were loaded to a 96-well microplate; 300 μL of Reagent 1 was added and incubated for 3 min at 37°C, then 150 μL of Reagent 2 was added for 4.5 min at 37°C. The optical absorbance was read at 550 nm.

### Statistical analysis

Each dietary and nutraceutical intervention group contained a minimum of eight mice, which was predicted to provide sufficient power based on the coefficient of variation of the key measures previously published [9,11,12,17,18]. All the statistical analyses were conducted by one-way ANOVA analyses followed by Tukey’s post hoc tests. The Pearson’s correlation coefficient describing the association between parenchymal protein extravasation (IgG and apoB) and parenchymal inflammatory markers, or plasma oxidative markers was determined. Statistical significance was considered at *P* <0.05. Data were expressed as means ±SEM.

## Results

### Mice have similar weight gain pattern and plasma lipids

Animals maintained on the diets enriched in SFA and diets supplemented with nutraceutical agents were well tolerated. The total calories consumed were approximately equivalent for the mice maintained on LF control chow and high SFA diet, and a similar weight gain was recorded in most treatment groups for a nine-month intervention period (Table 1). However, the final mean weight of the SFA+ALA group of mice was less than the SFA group (*P* < 0.05).

**Table 1:** Mean weight and weight gain

	**LF**	**LF+GEA**	**LF+ALA**	**LF+Niacin**	**LF+NA**	**SFA**	**SFA+GEA**	**SFA+ALA**	**SFA+Niacin**	**SFA+NA**
**Final mean weight (g)**	23.36	25.01	21.71	22.81	23.16	23.56	24.95	20.51*	23.16	22.13
**SEM**	0.3995	0.7448	0.2806	0.6906	0.5106	1.154	0.4088	0.4228	0.2275	0.4956
**Mean weight gain (g)**	9.05	9.26	5.26	7.78	7.27	8.25	9.76	4.85	7.96	7.29

The mean weights with standard error of mean at the end of nine months dietary intervention are shown in low-fat (LF), saturated fat (SFA) or diets supplemented with nutraceutical aged garlic extract-aged (+GEA), alpha-lipoic acid (+ALA), niacin (+Niacin), or nicotinamide (+NA) groups. The differences of means from the beginning to the end of dietary intervention in each group are also shown. *Indicates the statistical significance at *P* < 0.05 compared to SFA group by using one-way ANOVA followed by Tukey’s post hoc test.

**Figure 1:**
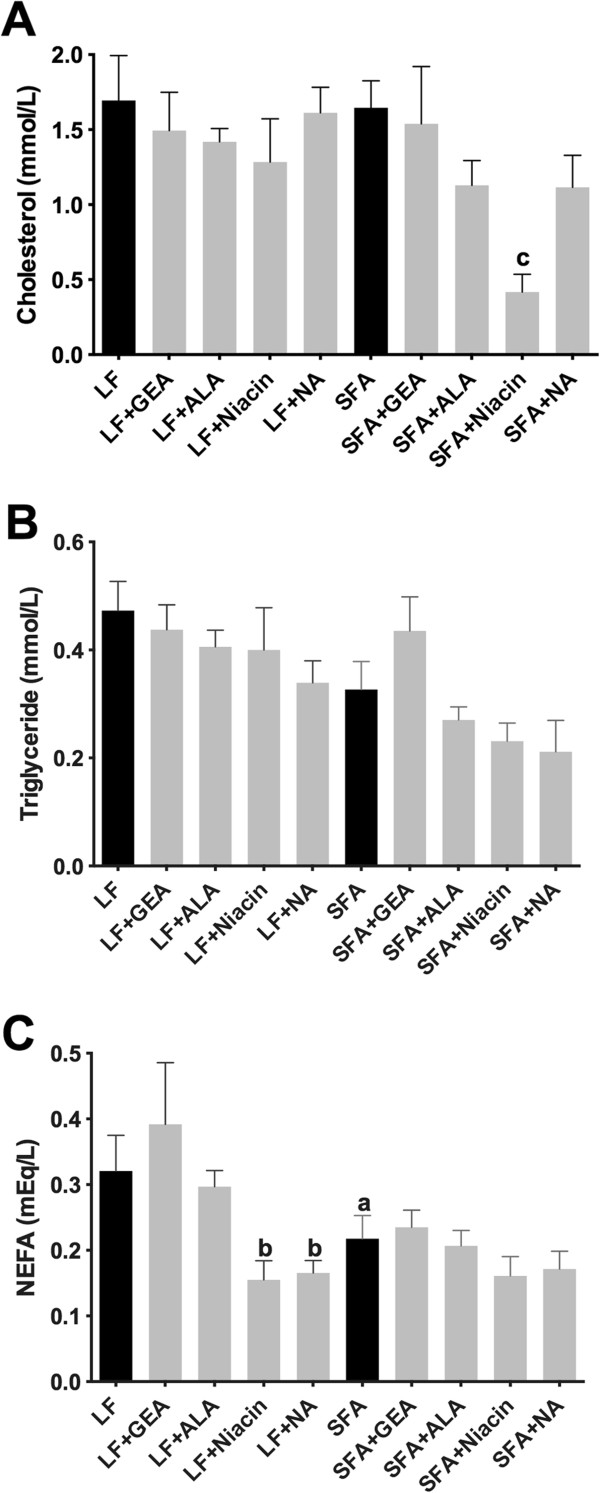
**Plasma lipids.** The plasma concentrations of cholesterol (**A**), triglyceride (**B**), and non-esterified fatty acid (NEFA) (**C**) were analyzed with commercially available colorimetric kits in mice maintained on low-fat control chow (LF), diet enriched in saturated fat (SFA) or each diet supplemented with nutraceutical garlic extract-aged (+GEA), alpha-lipoic acid (+ALA), niacin (+Niacin) or nicotinamide (+NA) for nine months. One-way ANOVA followed by Tukey’s post hoc test was used to analyze the statistical significance at *P* <0.05. (**a**) Significant difference between LF and SFA; (**b**) Significant difference between LF and each nutraceutical treatment; and (**c**) Significant difference between SFA and each nutraceutical treatment.

The plasma concentration of cholesterol and triglyceride of mice maintained on an SFA diet was not significantly different from that of mice maintained on an LF diet (Figure 1A and B). However, the plasma concentration of NEFA was significantly reduced in SFA fed mice compared to LF fed mice (*P* < 0.05) (Figure 1C). Supplementation of niacin in SFA diet significantly reduced plasma cholesterol, whereas no difference was found in LF+niacin group. Both niacin and NA supplementation significantly lowered the plasma NEFA concentration in the LF mice.

### Chronic ingestion of SFA diet causes severe breakdown of BBB, but BBB integrity is restored by nutraceutical agents

**Figure 2:**
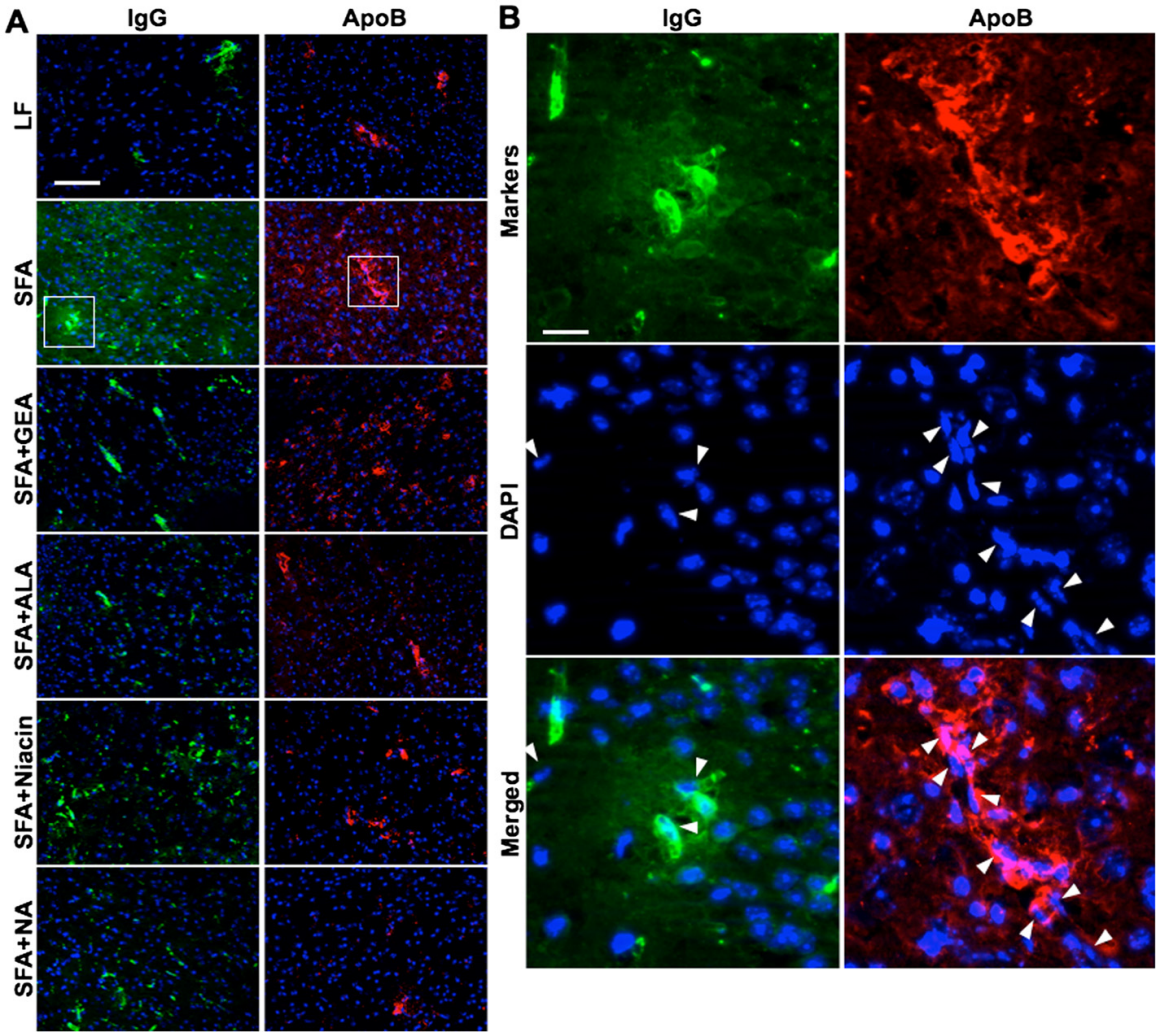
**Immunofluorescent micrographs of cerebral extravasations of plasma IgG and apolipoprotein B lipoproteins.** The integrity of the BBB was assessed by the detection of brain parenchymal extravasation of plasma derived IgG, and apoB lipoproteins in mice maintained on low-fat control chow (LF), diet enriched in saturated fat (SFA) or each diet supplemented with nutraceutical garlic extract-aged (+GEA), alpha-lipoic acid (+ALA), niacin (+Niacin), or nicotinamide (+NA). (**A**) The representative images of cerebral extravasation of IgG or apoB in the cortex are shown in green or red, respectively. Nuclei are shown in blue. Scale bar indicates 100 μm. (**B**) The magnified images of the regions of interest are indicated by a white rectangle in frame A. Vascular endothelial cells are shown with white arrowheads. Scale bar indicates 12 μm.

**Figure 3:**
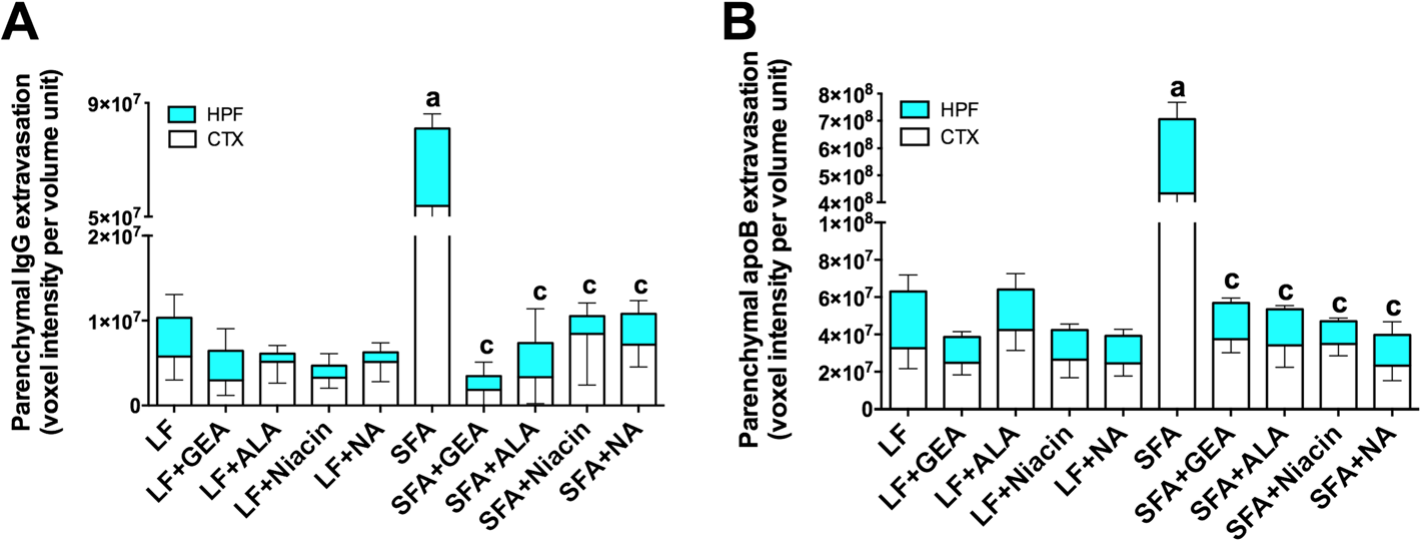
**3-D quantitative analyses of cerebral IgG and apoB extravasation.** The optical staining intensities of IgG (**A**) and apoB (**B**) were measured with 3-D image analysis software and shown in the cortex (CTX) and hippocampal formation (HPF) of mice maintained on low-fat control chow (LF), diet enriched in saturated fat (SFA) or each diet supplemented with nutraceutical garlic extract-aged (+GEA), alpha-lipoic acid (+ALA), niacin (+Niacin), or nicotinamide (+NA) for nine months. One-way ANOVA followed by Tukey’s post hoc test was used to analyze the statistical significance at *P* <0.05. Data were analyzed in cortex and hippocampal formation separately, however shown as combined since the statistical significance was identical. (**a**) Significant difference between LF and SFA; (**c**) Significant difference between SFA and each nutraceutical treatment.

We confirm substantial parenchymal perivascular abundance of plasma-derived proteins (IgG) and macromolecules (apoB lipoproteins) in mice maintained on SFA enriched diet for nine months (Figure 2). The 3-D quantitative immunomicroscopy showed that the parenchymal extravasation of IgG and apoB are substantially greater in SFA fed mice compared to mice maintained on LF control chow (Figure 3). However, provision of GEA, ALA, niacin, or NA with the SFA diet completely ameliorated the BBB damaging effect of dietary-SFA (Figures 2 and 3). The control mice maintained on LF diet for nine months showed some moderate signs of cerebral IgG and apoB lipoprotein extravasation (Figure 2 and 3), however this effect was not attenuated by co-supplementation with GEA, ALA, niacin, or NA.

### Parenchymal inflammations occur following the BBB disruption

**Figure 4:**
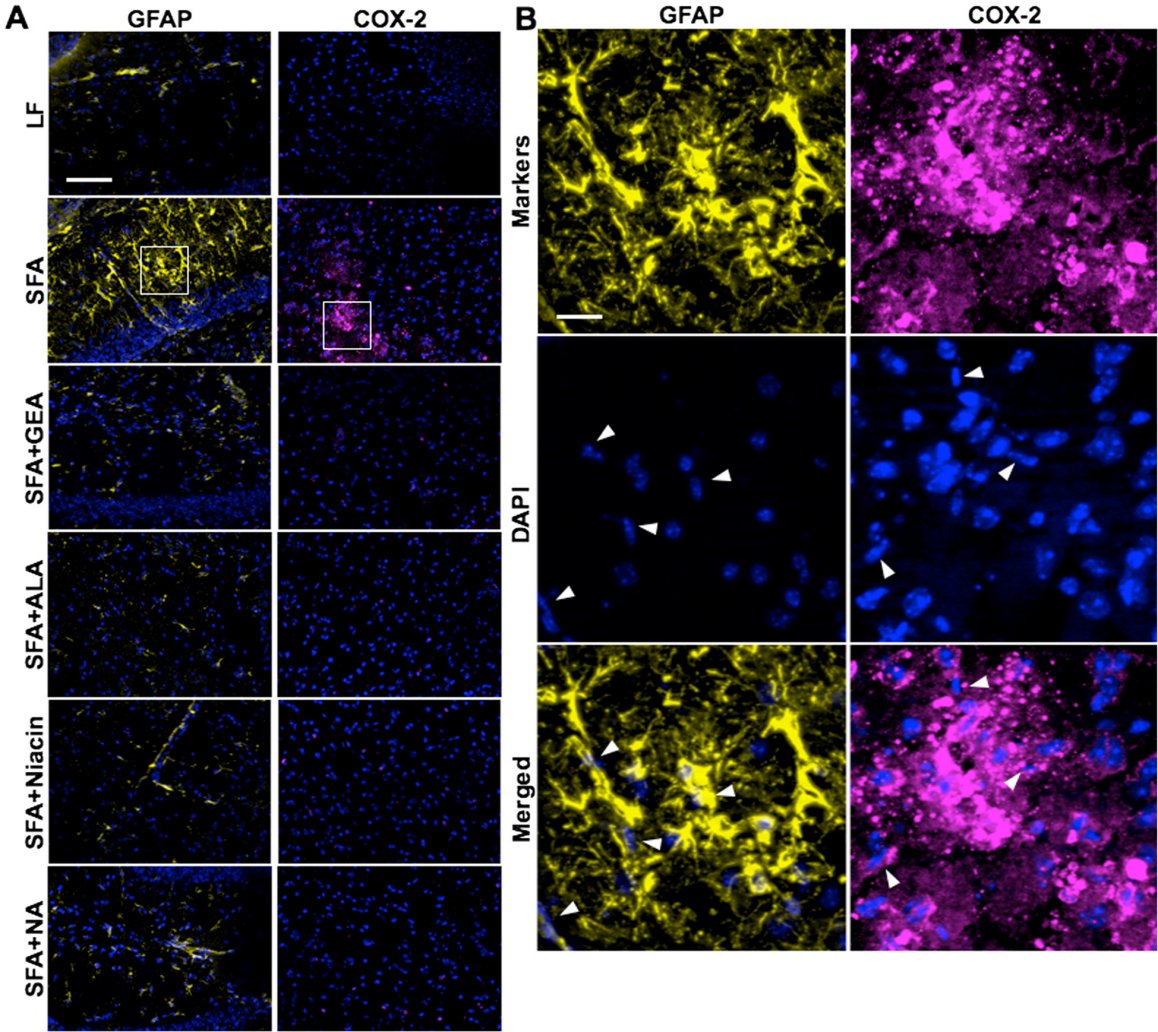
**Immunofluorescent micrographs of cerebral inflammation (GFAP) and oxidative stress (COX-2).** Cerebral astrocytic activation and oxidative stress were analyzed by the detection of parenchymal GFAP and COX-2 expression after nine months of dietary intervention with low-fat control chow (LF), a diet enriched in saturated fat (SFA) or LF/SFA diet supplemented with nutraceutical garlic extract-aged (+GEA), alpha-lipoic acid (+ALA), niacin (+Niacin), or nicotinamide (+NA). (**A**) The representative staining of GFAP in the hippocampus and COX-2 in the cortex are shown in yellow and cyan, respectively. Nuclei are shown in blue. The scale bar indicates 100 μm. (**B**) The magnified images of the regions of interest are indicated by a white rectangle in frame A. Vascular endothelial cells are shown with white arrowheads. The scale bar indicates 12 μm.

**Figure 5:**
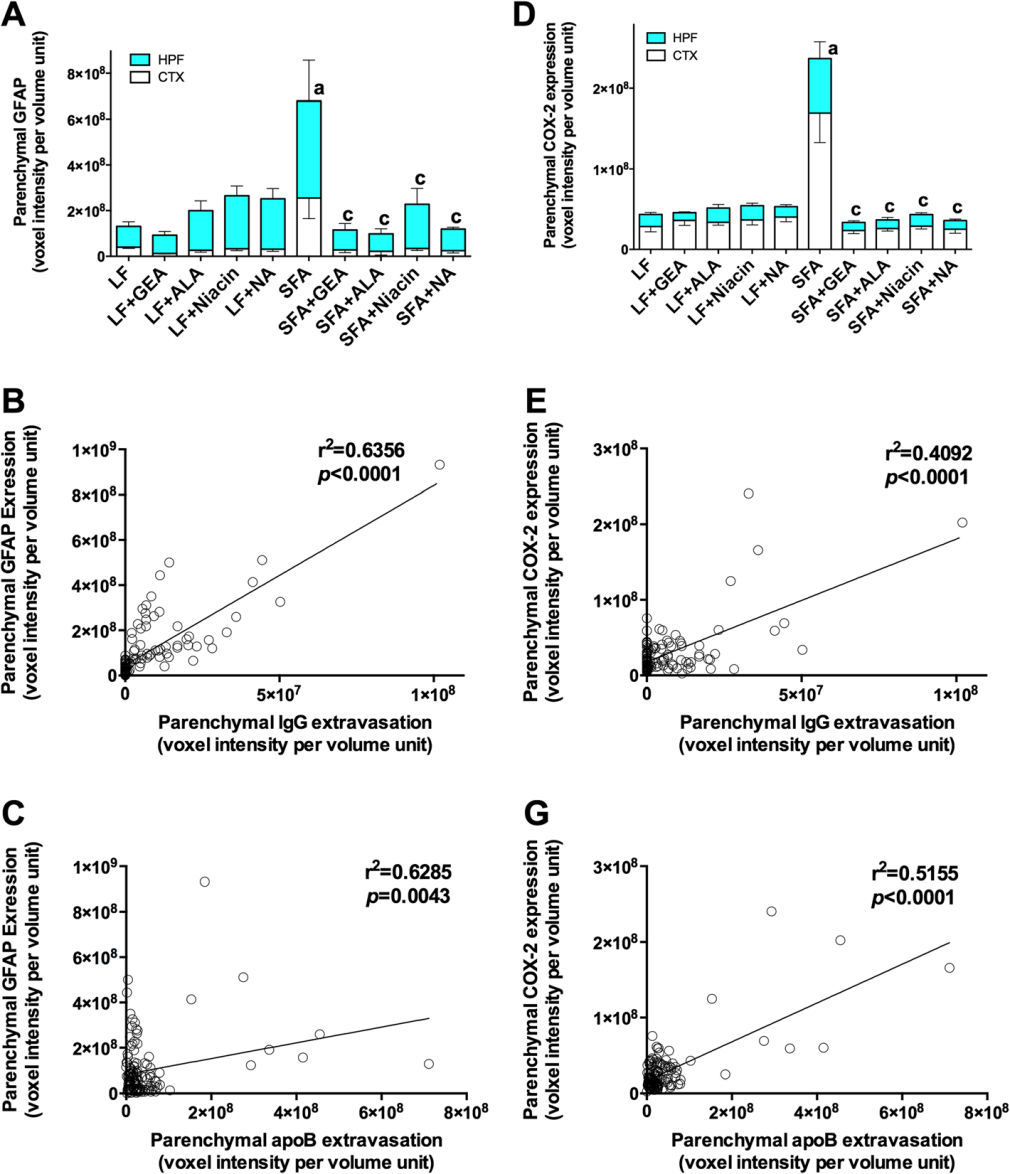
**3-D quantitative analyses of cerebral GFAP and COX-2 expressions.** The optical staining intensities of GFAP (**A**) and COX-2 (**D**) were measured with 3-D image analysis software and shown in the cortex (CTX) and hippocampal formation (HPF) of mice maintained on low-fat control chow (LF), diet enriched in saturated fat (SFA) or each diet supplemented with nutraceutical garlic extract-aged (+GEA), alpha-lipoic acid (+ALA), niacin (+Niacin) or nicotinamide (+NA) for nine months. One-way ANOVA followed by Tukey’s post hoc test was used to analyze the statistical significance at *P* <0.05. Data were analyzed in the cortex and hippocampal formation separately, however they are shown as combined since the statistical significance was identical. (**a**) Significant difference between LF and SFA; (**c**) Significant difference between SFA and each nutraceutical treatment. (**B** and **E**) show the correlation between parenchymal IgG extravasation and GFAP or COX-2 expression in all groups. Pearson’s correlation coefficient is indicated. For GFAP and parenchymal abundance of plasma proteins (**B** and **E**) and for COX-2 versus IgG and apoB (**C** and **G**).

Evidence of significant neurovascular inflammation and oxidative stress was concomitant with compromised BBB integrity in mice maintained on an SFA enriched diet for nine months. Cerebral parenchymal abundance of GFAP was elevated 3-fold in SFA mice compared to LF-fed mice at nine-months (Figure 4). Similarly, COX-2 expression was substantially increased because of chronic ingestion of the SFA-enriched chow (Figure 4). Consistent with the suppression of parenchymal plasma protein extravasation in SFA mice, provision of either GEA, ALA, niacin, or NA, completely suppressed the SFA-induced expression of GFAP and COX-2 (Figures 4 and 5A,D). A heightened state of neurovascular inflammation was not indicated by GFAP and COX-2 in mice maintained on the LF-diet for nine months (Figures 4 and 5A,D). A causal association between inflammation (indicated by GFAP and COX-2 abundance) and BBB-dysfunction (parenchymal IgG and apoB lipoprotein abundance) is suggested by Pearson’s correlation analysis (Figure 5B,C,E,F).

### Nutraceutical agents restore the systemic oxidative status

**Figure 6:**
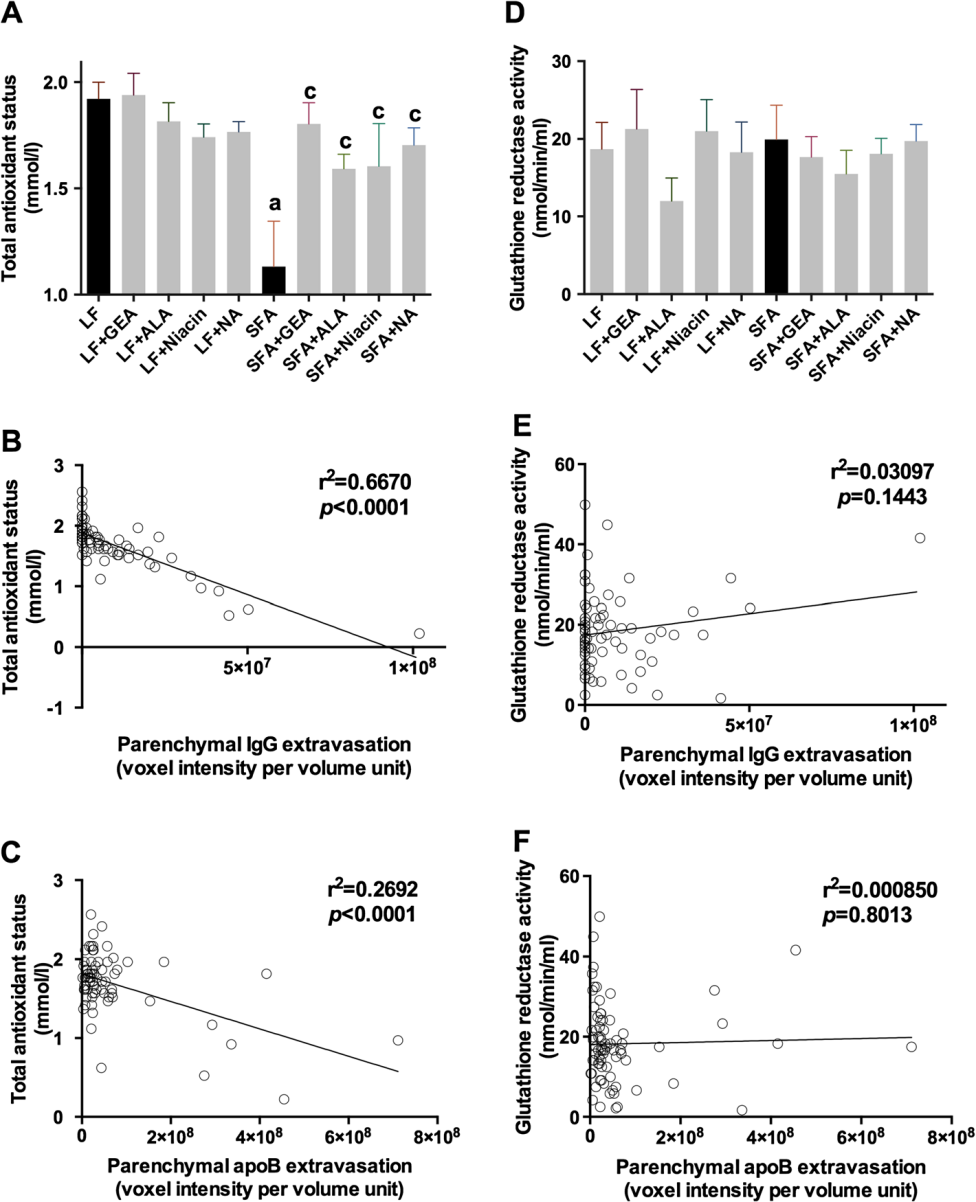
**Plasma anti-oxidant status.** The plasma total anti-oxidant status (**A**) and activity of glutathione reductase (**D**) were measured with commercially available colorimetric kits in mice maintained on low-fat control chow (LF), diet enriched in saturated fat (SFA), or each diet supplemented with nutraceutical garlic extract-aged (+GEA), alpha-lipoic acid (+ALA), niacin (+Niacin), or nicotinamide (+NA) for nine months. One-way ANOVA followed by Tukey’s post hoc test was used to analyze the statistical significance at *P* <0.05. (**a**) Significant difference between LF and SFA; (**c**) Significant difference between SFA and each nutraceutical treatment. (**B** and **E**) show the correlation between parenchymal IgG extravasation and total anti-oxidative status or glutathione reductase activity in all groups. The correlation coefficient was analyzed by Pearson’s analysis. Similarly, (**C** and **F**) show Pearson’s correlation coefficient between parenchymal apoB extravasation and total anti-oxidative status or glutathione reductase activity in all groups.

Plasma total anti-oxidative status was expressed relative to *in vitro* suppression of enzymatic genesis of a cation radical. Plasma TAS was significantly less in SFA fed mice compared to the LF fed control (Figure 6A). In contrast, mice supplemented with either GEA, ALA, niacin, or NA in the SFA treatment groups had a plasma TAS similar to age-matched low-fat controls. Pearson’s correlation analysis showed that the plasma TAS was negatively associated with parenchymal IgG and apoB lipoprotein extravasation in mice (Figure 6B,C). Glutathione reductase activity was not reduced in SFA mice compared to controls and provision of nutraceuticals had no significant effect (Figure 6D). Glutathione reductase activity was not associated with cerebral IgG or apoB-lipoprotein extravasation (Figure 6E,F).

## Discussion

The present study investigated the efficacy of the anti-oxidant nutraceuticals GEA, ALA, niacin, and NA in preventing BBB disruption in genetically unmanipulated mice maintained on a pro-inflammatory diet enriched in SFA. Consistent with previous studies showing progressive deterioration of BBB commensurate with the duration of feeding [7,9,11,12,17,18], in this study mice maintained on the SFA diet for nine months had substantial breakdown of BBB function. Compromised BBB integrity was indicated by established methodologies which considered the parenchymal abundance of plasma derived IgG, and also by the extravasation of larger macromolecules (apoB triglyceride rich lipoprotein) [9,11,12,17,18,37,38]. Quantitative 3-D immunohistochemical microscopy indicated that cerebral extravasation of IgG and apoB were substantially greater in the mice maintained on the SFA diet for nine months compared to the control LF-fed mice. Moreover, the dietary SFA-induced blood-to-brain kinetics and retention of plasma proteins and macromolecules occurred concomitant with evidence of heightened state of inflammation (GFAP and COX-2). Indeed, the correlation analyses showed an association between inflammation and parenchymal IgG and apoB abundance. In addition, our recent study showed the colocalization of parenchymal IgG extravasation and increased GFAP abundance in the same SFA-fed mouse model [12]. Interestingly, provision of the anti-oxidants GEA, ALA, niacin, or NA showed similar effects and completely suppressed SFA-induced BBB dysfunction. The prevention of BBB disruption by the indicated nutraceuticals occurred concomitant with the suppression of inflammation (GFAP and COX-2) and restoration of plasma total anti-oxidative status.

Earlier studies suggested that dyslipidemia might be central to brain capillary disturbances in this established model of BBB dysfunction [9,39,40]. In the present study, SFA feeding for nine months had no significant effect on the plasma concentration of cholesterol, triglycerides, or NEFA. Cell culture studies suggest several mechanisms by which dietary cholesterol may be pro-inflammatory and some of these appear to be analogous to the effects of dietary SFA. Yao and colleagues reported that excess cholesterol causes ER and mitochondrial stress that can lead to apoptosis [41]. Central to the latter, may be regulatory processes via altered ceramide/sphingosine-1-phosphate homeostasis [42]. Interestingly, Myriocin, an inhibitor of ceramide biosynthesis was effective in ameliorating ceramide accumulation in animals fed diets enriched in SFA [43,44]. Other mechanisms for SFA-induced alterations in the BBB functions include stimulation of NADPH-oxidase derived reactive oxygen species generated by activated microglial cells [45]. Substantially increased parenchymal COX-2 activity and diminished anti-oxidant status are consistent with exaggerated oxidative metabolism. COX-2 is an inducible enzyme most commonly associated with activated macrophages [46]. Increased abundance of COX-2 in SFA mice is consistent with the pro-inflammatory effects of SFA reported in many other studies [47,48]. Whilst not directly explored in this study, COX-2 may be associated with activated glial cells in mice fed SFA. Moreover, GFAP, a hallmark feature of activated glial cells, was markedly greater in the SFA treatment group suggesting the heightened cerebrovascular inflammation. In addition, our previous study also showed elevated peri-cerebrovascular CD68 immunoreactivity in SFA-fed mice [39]. Although markers of systemic inflammation were not measured in the present study, glutathione reductase, which serves to recycle glutathione by reducing its oxidized form (glutathione disulfide), was not significantly different in SFA mice, suggesting that this pathway for minimizing oxidative stress was not compromised in SFA-fed mice [49]. Many studies provide evidence that redox (reduction-oxidation) homeostasis is associated with vascular integrity via an axis of exposure to potentially toxic peroxides and free radicals [50-52]. Relevant to the findings described, a study by Kumar et al. reported that elevated plasma malondialdehyde and nitrate/nitrite were positively associated with increased BBB permeability in subjects with perinatal asphyxia [50]. A recent study reported that a high-fat diet (58% fat of total energy) significantly increased BBB permeability concomitant with compromised anti-oxidative status [7].

A clinical study by Williams et al. showed that GEA improved endothelial function in subjects with cardiovascular disease without altering plasma lipid homeostasis [53]. In insulin resistant subjects, ALA reduced the systemic oxidative stress and improved vascular endothelial function whilst having no effect on plasma lipids or lipoproteins [54]. ALA is transported through the BBB and reported to suppress cerebral and peripheral inflammation and oxidative stress [55]. Animal studies report that GEA attenuates systemic and central inflammation through increased hemoxygenase-1 activity and superoxide scavenging, and through decreased superoxide production both *in vivo* and *in vitro*[56,57]. In rats with subarachnoid hemorrhage, ALA supplementation completely suppressed the BBB disturbances, reduced parenchymal neuroinflammation, and increased plasma anti-oxidative status [58]. In three alternate animal models of systemic inflammation, ALA was shown to reduce cerebral reactive oxygen species and GFAP expression via increased cerebral superoxide dismutase, glutathione, and glutathione peroxidase activity [59-61]. Collectively, the studies suggest that GEA and ALA are likely to protect BBB function by suppressing systemic and central oxidative stress and inflammatory pathways.

In this study, supplementation of niacin or NA significantly reduced plasma cholesterol concentration in SFA-fed mice. These two agents also reduced the plasma concentration of NEFA in the LF-control group. Maintenance of BBB function in mice given niacin or NA may have in part reflected diminished vascular exposure to plasma lipids; however, this is unlikely given that the SFA fed group of mice were normolipidemic. Both niacin and NA at the dose rates indicated were equipotent to GEA and ALA in suppressing cerebral neuroinflammation (GFAP and COX-2) and oxidative homeostasis (plasma oxidative status). Ganji et al. showed in vascular endothelial cells *in vitro*, that niacin significantly increased cellular NADPH and glutathione levels, and reduced reactive oxygen species production and low-density lipoprotein oxidation [62].

A modest increase in cerebral extravasation of plasma IgG and apoB was observed in the LF-treatment group at nine months. The findings are consistent with an aging effect on BBB function recently reported in the same model, commencing at 30 weeks of age [12]. We extend on those findings and show that the supplementation of anti-oxidative nutraceutical agents did not prevent these aging-related alterations to BBB function. This finding is perhaps not unexpected in the context that TAS was similar to the LF control group. The findings suggest that the aging-related alterations in BBB function may occur independent of oxidative stress pathways [63-65].

## Conclusions

The primary findings of this study showed that compromised systemic anti-oxidative status induced by chronic SFA diet ingestion was associated with BBB dysfunction and neurovascular inflammatory responses. The provision of the anti-oxidants GEA, ALA, niacin, and NA prevents disruption of the BBB in high SFA-fed mice, concomitant with an improved redox state. However, the indicated nutraceuticals had no beneficial effects on aging-related disturbance in BBB function.

## Abbreviations

ALA: Alpha lipoic acid; apoB: Apolipoprotein B; BBB: Blood–brain barrier; COX-2: Cyclooxygenase-2; GEA: Garlic extracts-aged; GFAP: Glial fibrillar acidic protein; IgG: Immunoglobulin G; LF: Low-fat; NA: Nicotinamide; NEFA: Non-esterified fatty acids; SFA: Saturated fatty acids; TAS: Total anti-oxidant status.

## Competing interests

The authors declare that they have no competing interests.

## Authors’ contributions

RT conceived and designed the study and was involved in sample and data collection, data interpretation, and manuscript preparation. MMPG, VL, and CG were involved in sample and data collection. JCM was responsible for the supervision of the entire project and was involved in the study design, data interpretation, manuscript preparation, and funding. All authors read and approved the final manuscript.

## Author’s information

RT is a Research Fellow of the National Health and Medical Research Council of Australia and of School of Public Health, Faculty of Health Sciences, Curtin University. MMPG, VL, and CG are PhD candidates of the School of Public Health, Faculty of Health Sciences, Curtin University, supervised by RT and JCM. MMPG, VL, and CG are supported by the Australian Postgraduate Awards scholarship. JCM is a Professor of Faculty of Health Sciences, Curtin University, and a Director of Centre for Metabolic Fitness, Australian Technology Network.
